# Partnering With Youth to Enhance Healthcare Access and Experience: Lessons Learned From a Teen Advisory Group

**DOI:** 10.1111/hex.70220

**Published:** 2025-03-23

**Authors:** Christi H. Esquivel, Sara A. Flores, Kristen Garcia, Whitney Garney, Kelly Wilson

**Affiliations:** ^1^ School of Public Health Texas A&M University College Station Texas USA; ^2^ Telehealth Institute Texas A&M University College Station Texas USA; ^3^ School of Nursing Texas A&M University Bryan Texas USA

**Keywords:** health promotion, program development, Youth advisory board

## Abstract

**Introduction:**

Youth are experts of their experiences and well‐positioned to be effective partners in innovation. A common method to engage youth in innovation is through an advisory board. This paper focuses on the development and implementation of a nation‐wide teen advisory group (TAG) as part of the Comprehensive Healthcare for Adolescents Initiative (CHAI) Project at Texas A&M University. The CHAI project aimed to develop innovative programs that increase youth access to, and enhance experiences with, healthcare services. A TAG was developed at the project's onset to ensure youth voice and experiences drove the program development process. This paper describes methods used to recruit and convene CHAI TAG members, results and outcomes of their efforts throughout the program development process, and conclusions and lessons learned for practitioners and youth engagement researchers.

**Methods:**

Project staff from Texas A&M University recruited youth from across the nation through electronic mediums in Fall 2020. Engagement comprised meeting attendance and take‐home activities. All meetings and activities were optional based on the youth's availability.

**Results:**

Throughout the project, CHAI hosted 31 virtual meetings and offered nearly 20 unique activity opportunities for TAG members. Their insights and ideas drove the direction of the program development process, informing program design and content.

**Conclusion:**

The TAG's insights and feedback were instrumental in developing programs related to youth‐friendly spaces, confidentiality, assessing for unmet needs and healthcare navigation. The program's success in engaging youth provides a model for other youth engagement efforts.

**Patient or Public Contribution:**

This paper focuses on how a group of young people from across the country served as members of a teen advisory group (TAG) to develop innovative programs for healthcare settings. Said TAG members provided invaluable insight that informed and drove a cyclical program development process. The TAG members began by sharing their experiences with healthcare services and providers, then they envisioned ideal healthcare encounters. Such experiences, along with the youths' continuous feedback, led to the development and fine‐tuning of three program ideas focused on organizational change to improve adolescent healthcare.

## 1 Introduction

Innovation in health education and healthcare delivery is essential as the needs and interests of society continue to evolve. Traditional healthcare models often face limitations in addressing the intricacies of modern health issues, emphasizing the need for innovative strategies that go beyond routine practice [1]. Additional researchers [2] further emphasize the importance of a forward‐thinking approach to address the unique needs of diverse communities and to advance health equity. Fostering innovation becomes not only imperative, but also a cornerstone for developing effective health education programs and services that can navigate the complexities of health‐related needs and challenges.

Effective innovation requires intentional engagement of the priority population and associated stakeholders [3, 4]. By involving end‐users in the design process, programs and services can more effectively address the unique needs and preferences of diverse populations. Incorporating user feedback fosters an empathetic understanding of their experiences, enabling the creation of solutions that resonate with their lived realities [5]. Applying these human‐centred design principles places end‐users at the core of the development process and promotes iterative testing and program refinement, which ensures programs align with the dynamic and evolving landscape of healthcare [6]. This approach not only enhances the likelihood of user acceptance but also contributes to creating sustainable and adaptable health programs that more adequately meet the needs of the priority population(s) they aim to serve.

### 1.1 Adolescent Health

An individual's experiences and behaviours during adolescence are intimately associated with their lifelong health [7]. As the adolescent population in the United States continues to grow, so does the need to improve adolescent health service delivery [8]. Health services research has historically positioned youth as participants [9, 10]; however, there is a critical need to more actively engage youth, given the implications of involving end‐users in innovation.

One way to actively engage youth in innovation is through a youth advisory group. Establishing a youth advisory group is rooted in the ethos of patient‐centred care, where the youth actively participate in decision‐making as a means to tailor interventions to their specific needs [1]. This approach ensures inclusivity and responsiveness, as the youth bring forth unique insights into their health concerns and preferences. Moreover, the literature suggests that such engagement fosters a sense of ownership and empowerment among the youth, positioning them as active contributors rather than passive recipients of programs and services [11].

As organizations embark on the journey of establishing a youth advisory group, they must navigate the nuances of communication, power dynamics and cultural sensitivity to create an environment conducive to meaningful youth engagement [11]. This paper focuses on the process of developing and implementing a teen advisory group, including outcomes, benefits and challenges, from an organizational perspective. The project's success in engaging youth provides a model for other youth engagement efforts. To adequately share the teens' perspectives, a second paper is in development focusing on youth participant reflections and descriptions of their experiences.

### 1.2 TAG: Mission and Goals

**Figure 1:**
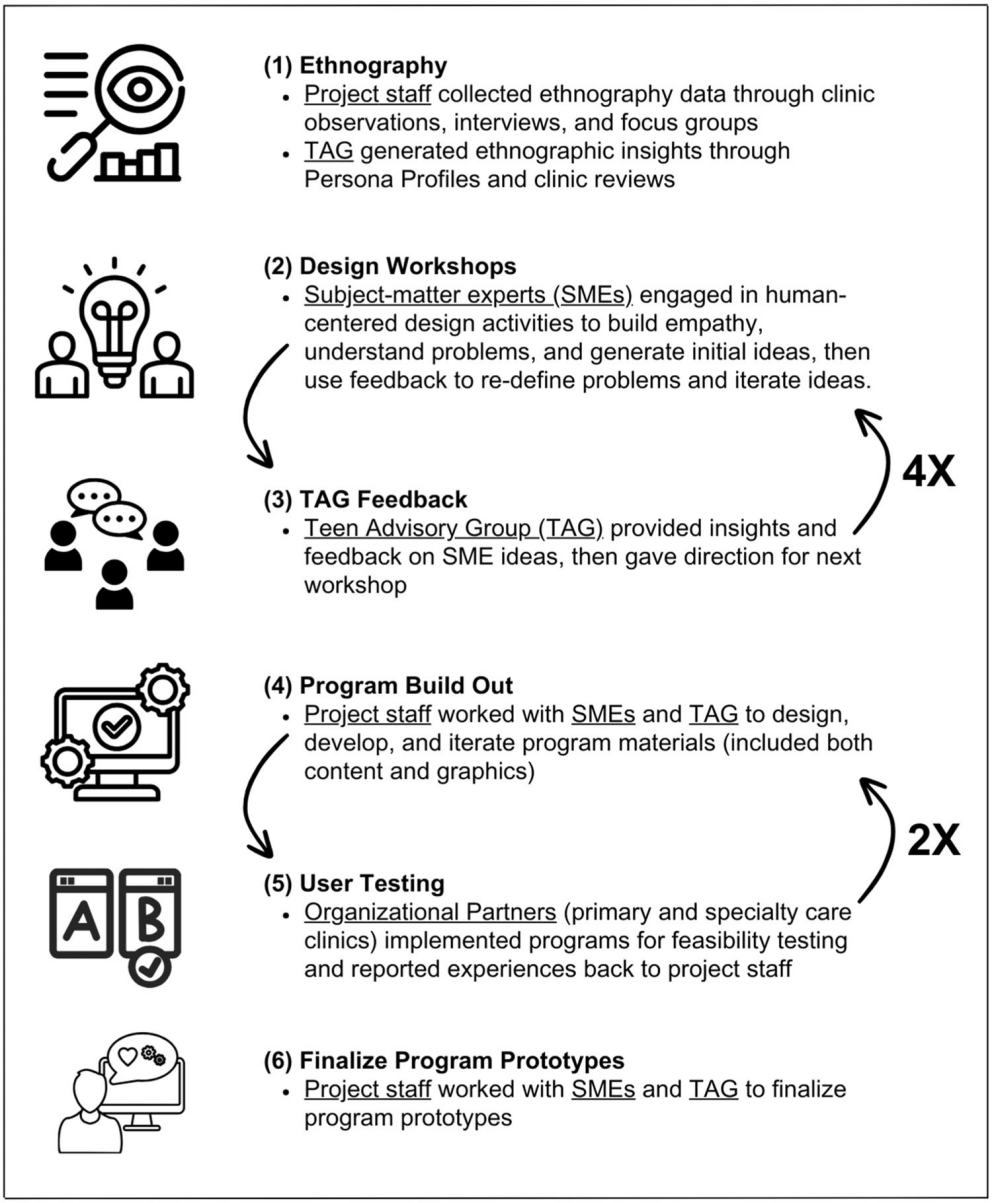
Program development process.

The Comprehensive Healthcare for Adolescents Initiative (CHAI) Project at Texas A&M University focused on developing innovative programs that increase youth access to, and enhance experiences with, healthcare services. A network of subject‐matter experts (SMEs) brainstormed program ideas and prototypes over 5 design sprint sessions. A national teen advisory group (TAG) was established to ensure youth voice and experience was meaningfully integrated into this process. The TAG convened after each design session to offer feedback on the acceptability of program ideas and share relevant healthcare experiences, which provided direction for future sessions. The TAG was formed to (1) share insights and experiences; (2) generate ideas and prototypes; (3) test prototypes; and (4) provide feedback on ideas and prototypes. The back‐and‐forth structure of meeting with the TAG in between sessions with SMEs allowed the youth to have a more pivotal role in the program development process. See Figure 1 for a graphic representation of the overarching program design process. The process for identifying the core challenge and exploring and developing innovative programs was informed by the Framework for Public Health Innovations [12].

## 2 Methods

### 2.1 Recruitment, Selection and Membership

TAG recruitment began in September 2020. Texas A&M University staff members that were part of the CHAI team emailed announcements containing digital flyers to youth‐serving organizations and professionals across the United States in an effort to recruit a diverse, nation‐wide sample. Flyers were also distributed to stakeholders who expressed interest after hearing about the opportunity. All stakeholders were asked to share the flier and opportunity with at least 1–2 youth. The flier was brief and included a short URL and QR code that linked youth to a website with more information about the advisory group including (1) a description of the CHAI project, (2) the purpose of the advisory group, (3) anticipated activities and tasks for advisory group members and (4) information on how to apply. Eligible participants included English‐speaking youth ages 14 and up, living in the United States with access to the internet during meeting hours. Such requirements afforded a large number of youth to be eligible.

The application process comprised two parts. First, interested youth completed an electronic form containing questions about them and their interest in the group (see Table 1). Then, the CHAI staff leading the advisory group made a video to send out to the applicants. The video introduced the staff members and included a prompt for part two of the application, which entailed youth showing or telling staff more about their ideas for the future of healthcare. Youth were asked to describe their *ideal* healthcare experience—what it looks like, how it makes them feel, and what happens during the visit. Youth could submit this in whatever form they preferred including but not limited to a picture, collage, video, poem or other text/narrative‐based format. Youth under the age of 18 were also required to submit a signed permission slip from an adult guardian.

**Table 1:** Advisory group application topics and questions.

**Contact Information** 1. Name 2. Age 3. City and state you live in 4. Grade level 5. Phone number 6. Email address 7. Best way to contact you
**About the Youth** 1. What is one of the biggest issues you think young people face with healthcare? 2. How can you help inform or change the future of healthcare? 3. If you could travel to any city, where would you go? 4. If you could have an endless supply of any food, what food would you pick? 5. What do you want to be/do when you grow up? 6. What are your interests and hobbies? 7. What is your favourite colour?
**Background and Demographics** 1. Race/Ethnic identity 2. Gender identity 3. Ever been to the doctor 4. Health insurance status 5. Access to computer or tablet with internet connection 6. Pregnant/parenting status

The CHAI team set minimal requirements around membership in effort to retain as many members as possible. Members were asked to engage in 60–90 min virtual monthly meetings, participate in additional meetings and events as they arose (optional and upon availability), complete some tasks on their own time and submit back to CHAI staff, and complete online trainings they were interested in. Members were not required to commit to anything, and were paid for every meeting, activity, etc., they participated in or completed. This flexibility allowed youth to be engaged as much or as little as they felt aligned with their schedule. Payments averaged to $25 per hour and were provided in the form of check or direct deposit, and gift cards; youth selected their preferred payment method for each payment period.

### 2.2 Meetings and Activities

**Figure 2:**
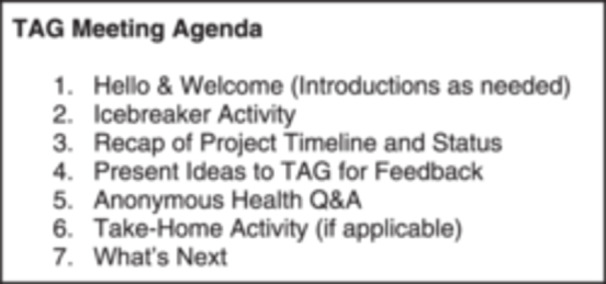
TAG meeting agenda.

Meetings occurred monthly from November 2020 to May 2023. Duplicate meetings were conducted on different days each month via Zoom to accommodate the various availability and time zones of TAG members. All meetings took place on weekday evenings (i.e., outside traditional work hours). This allowed members to attend whichever meeting best fit their schedule. All meetings were facilitated by two CHAI staff members and followed a similar agenda/format (see Figure 2 for a sample agenda). However, the purpose of meetings evolved throughout the project. Early meetings focused on gathering ethnographic insights from the TAG on youth experiences with healthcare services. Over time, meetings shifted to reviewing and iterating program ideas developed by SMEs, to reviewing and developing content and graphics for program prototypes. Later meetings focused on professional development sessions for the TAG members. A few in‐person design sessions (*n* = 5) were hosted in various cities home to multiple TAG members.

The CHAI project aimed for TAG to be mutually beneficial. First, the TAG would focus on CHAI‐related efforts and tasks. Small and large group activities were completed using interactive platforms such as Mural or Google Docs. Such platforms allowed for real‐time collaboration among the TAG members, which was particularly helpful when brainstorming and reiterating program ideas. All meetings were structured with opportunities for TAG members to share ideas out loud, one at a time, leaving time and space for all willing voices to share and be heard. There was a shared understanding that no experiences, ideas or feedback would be judged, belittled or dismissed, which encouraged engagement. Towards the end of each meeting, the TAG steered the direction of conversation based on health‐related topics and questions they wanted to discuss. Using a Google Form, TAG members submitted anonymous feedback, recommendations and health‐related questions to be answered aloud for everyone by trained health educators on the CHAI staff.

Between meetings, TAG members were often tasked with a creative assignment, such as developing Persona Profiles that reflect youth in their communities [13], creating TikTok videos that critiqued clinic environments, writing scripts for healthcare providers, drafting graphics for partner clinic spaces, and editing content for program materials. Participants were also offered access to virtual, asynchronous trainings to enhance their skills for innovation (topics: human‐centred design and systems thinking). Each activity tied directly to the goal of the CHAI project.

### 2.3 Data Collection and Analysis

Throughout the project, the CHAI team collected and analysed various forms of data (see Table 2). While some data sources did not centre on the TAG, all sources included questions that inquired about the cyclical nature of the TAG's involvement in the project.

**Table 2:** Data tools and descriptions.

Source	Purpose
**Design session surveys**	SMEs completed a survey after each design session to reflect on the process and activities, including TAG feedback.
**Interviews**	SMEs were interviewed at the end of the design sprint process to reflect on the cyclical process and strategies used to propel program development efforts.
**Design sprint after‐action reports**	The CHAI team documented design activities (including challenges and outcomes), key points of discussion, pivot points and next steps.
**TAG attendance and activity logs**	The CHAI team kept track of convening attendance (for meetings and events) and activity completion for all TAG members.
**TAG meeting minutes**	The CHAI team took meeting minutes for each TAG convening to document activities, key discussion points and feedback, pivot points, requests and recommendations.

All data sources were analysed thematically as archival documents. The evaluation team utilized open‐coding to identify emerging themes [14, 15]. All members of the team analysed all data sources independently to identify units of data and organize by common theme [16]. After analyzing the data independently, the evaluation team met to review the codes and identify key themes. The team triangulated the data across sources to look for patterns and key themes, which are discussed in the following section.

## 3 Results and Outcomes

The TAG's contribution exceeded its original intent and laid the foundation for various ideas to be explored and moulded into clinic‐based programs aimed at increasing access to, and enhancing experiences with healthcare services for adolescents. Key outcomes of the process used include (1) Attendance and engagement, (2) Meeting activities and purpose, (3) Authentic youth voice, (4) Youth‐Friendly Design and Content and (5) Challenges.

### 3.1 Attendance and Engagement

A total of 28 youths applied to be part of the TAG, and 19 agreed to membership and attended at least one meeting/event (see Table 3 for Demographics). CHAI staff hosted 31 virtual meetings over the course of 3 years. Engagement was tracked through meeting attendance, contribution to discussions, and activity completion. Members were encouraged to participate in as many sessions and activities as they wanted; with no minimum requirements. Attendance ranged from 3 to 16 members and averaged nine young people per meeting. TAG members earned $25 per meeting, with over $7500 being paid to young people for attendance. Facilitators offered the youth over 17 types of activities to complete *outside* of meetings. A range of 4–16 youths completed activities, with an average of seven young people completing each activity. Compensation for activities varied; over $5500 was paid to young people for completing activities outside of group meetings.

**Table 3:** TAG applicant demographics.

Race/Ethnicity		Gender	
Black or African American	45% (*n* = 13)	Female	79% (*n* = 23)
White or Caucasian	21% (*n* = 6)	Male	7% (*n* = 2)
Hispanic or Latinx	14% (*n* = 4)	Nonbinary	3% (*n* = 1)
Asian	7% (*n* = 2)	No response	7% (*n* = 2)
Multiracial	7% (*n* = 2)	**Age**	
American Indian or Alaska Native	3% (*n* = 1)	14	3% (*n* = 1)
No response	3% (*n* = 1)	15	28% (*n* = 8)
**Ever Been to Doctor**		16	31% (*n* = 9)
Yes	93% (*n* = 27)	17	34% (*n* = 10)
No	– (*n* = 0)	18	3% (*n* = 1)
No response	3% (*n* = 1)	**Region** *	
**Health Insurance Status**		South	66% (*n* = 19)
Insured	83% (*n* = 24)	West	24% (*n* = 7)
Uninsured	10% (*n* = 3)	Northeast	7% (*n* = 2)
Don't know	7% (*n* = 2)	Midwest	3% (*n* = 1)

* Regions are categorized using the US Census Bureau Regions and Divisions

### 3.2 Meeting Activities and Purpose

Over time, the purpose of TAG meetings and the subsequent activities evolved. During prototype testing phases, the project warranted less input and feedback from the TAG; however, the CHAI team wanted to maintain engagement with the TAG, and support their needs. Through emails and anonymous feedback/requests, the TAG requested professional development support, such as letters of recommendation and guidance on selecting a career path. TAG meetings then shifted to focus on professional development. This included workshops curated by CHAI included a series of guest speakers who shared their career paths and how they got to where they are now, followed by time for the TAG to ask questions. Several of the guest speakers were partnering SMEs. These meetings offered SMEs an opportunity to interact with the TAG and ‘give back’. Attendance records and meeting minutes indicated participation levels decreased during professional development‐focused meetings.

### 3.3 Authentic Youth Voice

The CHAI team at Texas A&M University, and partnering SMEs, viewed TAG members as experts of their own experiences regarding healthcare services and needs. They were encouraged to share as much as they were comfortable, and often solicited additional input from their peers in between meetings. Staff aimed to validate all experiences and ideas shared by TAG members. While the team would have liked for the TAG to meet and work directly with SME partners, the separation of groups ensured a more comfortable environment for the TAG to speak openly and honestly. It also allowed the staff to listen to their experiences and ask questions to further understand their experiences, rather than someone telling them ‘that wouldn't happen’. There were even times the TAG would submit video or audio recordings of their ideas and feedback so the SMEs could literally hear directly from young people. This was particularly impactful on the SMEs [17]. Several SMEs expressed that, although they understood youth needs, the TAG‐developed ethnography data improved their understanding of youth desires. One SME said, ‘It really resonated with me that kids want to be much more involved in their own care at every level. Maybe just reminding me of something that may have fallen into the background a little bit for me’. Another said, ‘What the youth liked was not completely aligned with what I was thinking. I found it helpful to hear what they were most excited about’.

SMEs found the TAG's feedback frustrating at times but continued to acknowledge its value. One SME said they noticed that the entire [ethnography] process ‘slowed everything down. And so that was in some ways like hard and uncomfortable for [them] to go that slowly, to watch things evolve and unfold’. However, they also admitted that the slow pace forced the design team ‘put [themselves’ in this person's shoes and think about every detail, from what they feel like when they approach your door, to the consent forms’. Another SME described how the TAG's feedback required them to recognize, ‘Flexibility in the design process can go a long way in helping you to achieve the impact that you want to have’. The same SME later said, ‘Some things that one youth really liked, another youth might not. But what was important was not listening to the “I like it” or “I don't like it”, but really the whys. And within the whys were opportunities to learn how to really iterate and innovate on an idea to allow it to have broader appeal’.

The TAG also provided insights and feedback through creative assignments, such as Persona Profiles and TikTok challenges. Creative assignments allowed for youth voices to authentically come through in their work and resulted in some unexpected, but positive effects on SMEs. One SME was inspired to revamp their own clinic space, using insights from the TAG and others elicited from their own youth advisory board. When recounting their experience, this SME said, ‘The things we got from that really changed our perceptions… The data they gave us has launched several initiatives [within the clinic] that we have now developed for not only youth and young adults, but also to increase our paediatric patient population’.

### 3.4 Youth‐Friendly Design and Content

The TAG contributed significantly to program design and the content within programs and program materials. Rather than having staff draft contents or materials, the TAG was often tasked to draft or review and edit program contents including graphics, patient‐facing materials, informational handouts, sample scripts, etc. This saved staff time and led to a variety of content to pull from for final program materials, and often led to having several samples in program packages for organizations to use. Most importantly, though, this resulted in program materials that were youth‐friendly, namely relatable, inclusive and relevant to their needs.

### 3.5 Challenges

As expected, there were several key challenges encountered throughout the project, including, but not limited to: competing priorities, staff time, and virtual engagement (see Table 4).

**Table 4:** Summary of challenges for engaging young people.

Challenge	Context	Attempted or Potential Solutions
**Competing Priorities**	Meetings occurred on weekday evenings, which tended to conflict with youth schedules, particularly after‐school activities and commitments.	Offer duplicate meetings allowing young people two days/times to attend. This also cut down on group size which allowed for more discussion.
**Staff Time**	Meetings required CHAI project staff to commit to working outside their traditional work hours (8 am–5 pm).	Offer staff comp time to offset the extra hours spent working outside their traditional schedules.
**Virtual Engagement**	(1) Virtual meetings often come with faster burnout and distracting environments. (2) It is difficult to foster a sense of community through online meetings. (3) Activities take longer virtually.	(1) Send members a description of meeting activities so they could be prepared as much as possible. (2) Begin with icebreakers and activities that allow small group/paired discussion. (3) Keep agendas as short as possible and build in ‘buffer’ time for activities.

## 4 Conclusions

The CHAI team adapted the TAG engagement process over the course of 3 years to ensure members' effectively contributed to the overall project through a mutually beneficial relationship. The successful process highlights two primary drivers of youth engagement: flexibility and creative assignments.

Flexibility served as a core tenant to the team's approach of engaging TAG members. Participation costs for youth in previous projects included heavy time demands, additional work burden and frustration with the process [18, 19]. To mitigate these costs, the project team offered flexibility in meeting days, meeting attendance, activity completion and expectations, styles of engagement, and compensation. This flexibility allowed young people to contribute as much or as little as they were able to, in a way that aligned with their schedules and other competing life priorities. Institutional policies limited forms of compensation; however, TAG members selected how they were paid for each invoice. Compensation options included direct deposit and checks, or electronic gift cards. The flexibility employed is consistent with accommodations described in the youth engagement literature [20, 21].

Creative assignments allowed youth to have fun while gathering and generating insights, and providing feedback. Stakeholder engagement should be as engaging and interactive as possible rather than asking questions and waiting for responses [20]. TAG members stated they had fun completing the creative assignments [17]. The progressive and interactive phases of the design thinking approach may have inspired creative confidence (i.e., the ability and courage to create innovative ideas) [22, 23, 24]. Creative assignments led to unexpected insights from the young people that may not have otherwise been revealed. Thus, creative assignments supported communication between the TAG and SMEs as well as SMEs' capacity to engage in critical reflection. SMEs appreciated the interactiveness of the insights and products that resulted from the creative assignments and were particularly impactful on their process. These mutual benefits underscore the importance of youth engagement in health services innovation [18, 25, 26, 27].

### 4.1 Organizational Benefits

The TAG offered many benefits to the project and organization including stronger appeal, increased innovation and improved trust. One noteworthy benefit was a heightened level of efficiency in the program development process. There's a phrase that is metaphorical to program development, ‘it's better to take an eraser to the drawing board, rather than a jack‐hammer to the concrete’. Convening the TAG after each design session allowed for continuous representation and input from young people, who were key stakeholders and users of the final programs. Their feedback allowed the design team to identify necessary pivots to program ideas in a timely manner before dedicating time to developing prototypes that might not resonate with young people. By directly involving young people in early program development efforts, organizations can avoid costly revisions later on, ultimately speeding up their timeline to launch programs and services.

### 4.2 Lessons Learned

The process of engaging youth through an advisory group format led to several lessons learned for future youth engagement. First, it is essential to meet consistently with youth—even if a project is at a place where there is not a lot of opportunity for the youth to actively contribute. It is still beneficial to hold meetings even if the sole purpose is for youth to connect with each other and build community. Second, unexpected insights came from creative assignments. During the TikTok challenge, youth were tasked to create video‐based reviews of health clinics, and other youth‐serving spaces. Unexpectedly, their feedback was on things the team would not have otherwise asked about including lighting/light fixtures, paint colours and excessive signs/promotional materials.

Fostering a sense of community through online‐only engagement is difficult. While the CHAI team intended for monthly meetings to be virtual, the team also intended to have in‐person meetings and events each year. However, due to COVID‐19, the TAG was unable to meet in‐person for the first 1.5 years of the project and were forced to only convene online. The online‐only format allowed the team to convene a more diverse group of young people representing various states and healthcare experiences in terms of physical and political environments. Moreover, the team took advantage of technological advances (i.e., digital visual collaboration environments) that support the four competencies of design thinking—critical thinking, creativity, communication and collaboration [28, 29]. A predominantly online format also may have aligned with teens’ preferences for socializing [30]. That said, the approach suffered from a lack of social presence and limited spontaneous conversation, which can pose challenges for fostering a sense of community [31, 32]. The TAG leadership allowed some time at the beginning of meetings for people to ‘network’ but should have built in more time and spent some meetings just focused on group forming. The CHAI team tried to foster community through icebreaker activities and small group breakout activities but would have benefitted from more conscious efforts around this. It is also worth considering hiring a youth engagement specialist to assist with starting the advisory group and facilitating the first few meetings.

## Author Contributions

**Christi H. Esquivel:** conceptualization, investigation, writing – original draft, methodology, validation, writing – review and editing, formal analysis, project administration. **Sara A. Flores:** resources, data curation, methodology, validation, visualization, writing – review and editing, writing – original draft, investigation. **Kristen Garcia:** validation, investigation, conceptualization, data curation, formal analysis. **Whitney Garney:** funding acquisition, supervision. **Kelly Wilson:** funding acquisition, supervision.

## Ethics Statement

The authors have nothing to report.

## Conflicts of Interest

The authors declare no conflicts of interest.

## Data Availability

The data that support the findings of this study are available on request from the corresponding author. The data are not publicly available due to privacy or ethical restrictions.
